# Saddle Pulmonary Embolism with Thrombus in Transit across a Patent Foramen Ovale

**DOI:** 10.1155/2017/6752709

**Published:** 2017-01-26

**Authors:** Fitzgerald Shepherd, Ashley White-Stern, Oloruntobi Rahaman, Damian Kurian, Karen Simon

**Affiliations:** ^1^Harlem Hospital Center, New York City, NY, USA; ^2^Columbia University Medical Center, New York City, NY, USA; ^3^Department of Cardiology, Harlem Hospital Center, New York City, NY, USA

## Abstract

This is the case of a 25-year-old obese man who presented with acute shortness of breath, chest pain, and palpitations. Of note, he lives a sedentary lifestyle and was recently hospitalized for incision and drainage of a left foot abscess. On presentation he was tachypnoeic, tachycardiac, and hypoxic but blood pressure was stable. Laboratory studies were significant for elevated D-dimer and mildly increased troponin. On further investigation he was found to have a saddle pulmonary embolism with massive clot burden. Echocardiogram revealed thrombus in transit and McConnell's sign. He underwent surgical embolectomy and closure of a patent foramen ovale. This is a particularly rare case, especially in such a young patient. Because this is a rare diagnosis, with insufficient data, there is no formally established treatment guideline. However, in patients who are good surgical candidates, studies have shown better outcome with surgical embolectomy as compared to anticoagulation alone or thrombolysis.

## 1. Introduction

Risk of deep venous thrombosis (DVT) is described succinctly by Virchow's triad: stasis, endothelial injury, and hypercoagulable state. The embolization of a DVT into the right heart predisposes to pulmonary embolism (PE). In the case of an embolic event, a patent foramen ovale (remnant of the fetal circulation) puts the patient at risk for paradoxical embolism.

## 2. Case Presentation

We report a case of an obese 25-year-old man, recently diagnosed diabetic, who presented to the emergency department with acute onset shortness of breath for several hours. Of note, he presented two weeks prior for left foot pain and was found to have an abscess on the plantar surface. He denied foot trauma, insect, or animal bites at that time. The abscess was successfully incised and drained, and he was admitted for intravenous antibiotics. During that admission he received subcutaneous heparin for DVT prophylaxis. Cultures grew methicillin-resistant* Staphylococcus aureus* (MRSA) and he was discharged five days later on culture-directed oral antibiotics. Since that time, he had been mostly sedentary but this was not a great departure from his typical habits.

The patient was at his usual state of health until the morning of presentation (while playing video games at home) when he had acute onset shortness of breath with associated palpitations and sharp left-sided chest pain. He had no fever, leg swelling, or calf pain. He denied smoking or illicit drug use. His symptoms continued and he was brought to the emergency department of his community hospital later that evening.

On physical examination, he was lying in bed slightly tachypnoeic at 20 breaths per minute but speaking in full sentences. He was tachycardiac at 135 beats per minute and afebrile at 97.1 degrees Fahrenheit and blood pressure was 144/96 mmHg. On cardiac examination, he had normal heart sounds with regular rhythm and no murmurs, rubs, or gallops. Breath sounds were vesicular on respiratory exam and no wheezes or crackles were appreciated. Extremities were without edema and the left foot wound was well healed.

**Figure 1:**
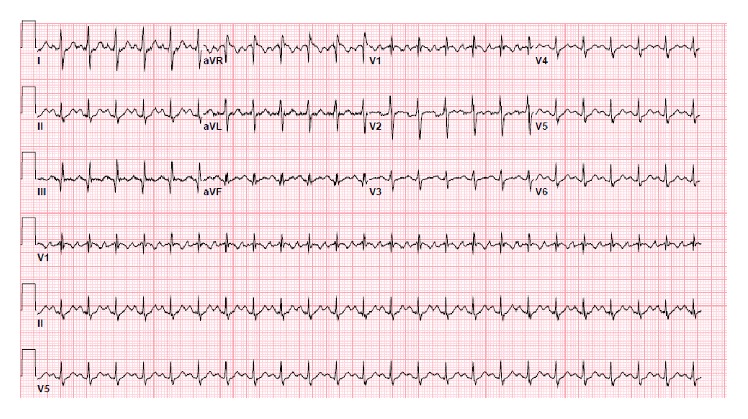
Electrocardiogram showing sinus tachycardia and right ventricular strain pattern S_1_Q_3_T_3_ (deep S wave in lead I, Q wave in lead III, and inverted T wave in lead III).

**Figure 2:**
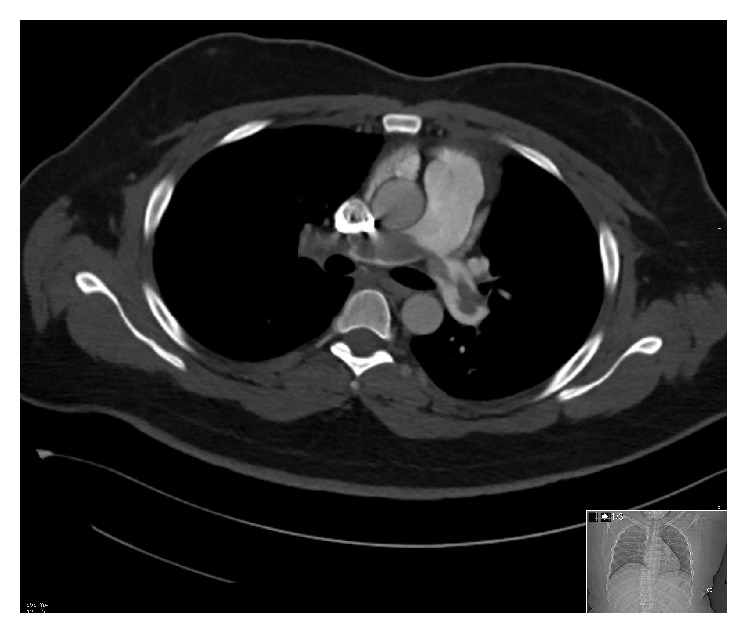
CT pulmonary angiogram revealed a saddle embolus extending into the main, lobar, and segmental pulmonary arterial branches bilaterally with massive clot burden and near complete occlusion of the right main and branch pulmonary arteries.

**Figure 3:**
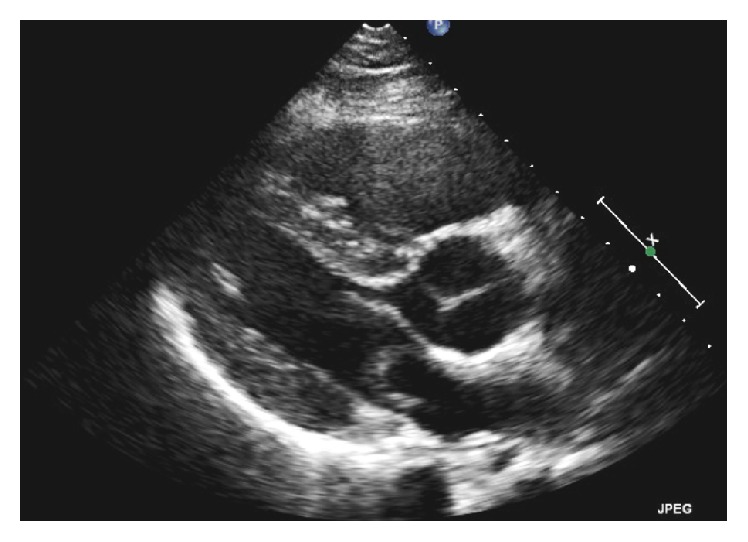
Echocardiogram showing mild concentric left ventricular hypertrophy and a serpentine mass in the left atrium, consistent with thrombus.

**Figure 4:**
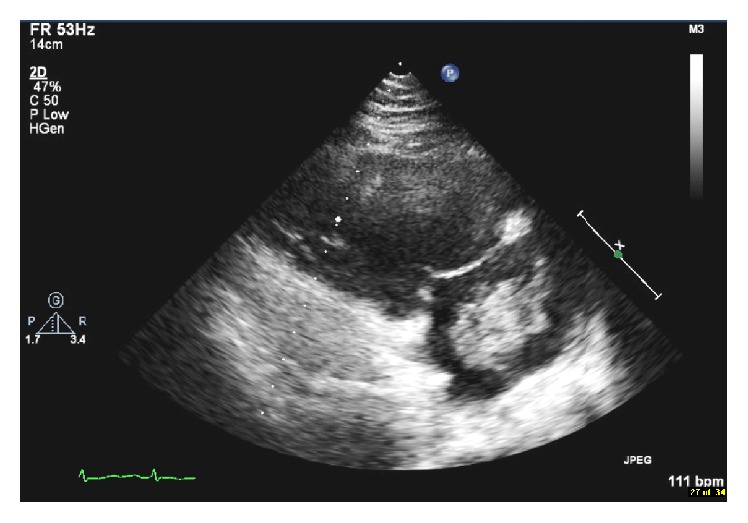
Echocardiogram showing large thrombus in the right atrium.

Laboratory results (Table 1) showed significantly elevated D-dimer and electrocardiogram showed sinus tachycardia with S_1_Q_3_T_3_ pattern (Figure 1). Given the suspicion for pulmonary embolism, he was presumptively started on low molecular weight heparin and computed tomography pulmonary angiogram was ordered (Figure 2). The radiologist urgently called in the early morning to give a preliminary report of an extensive saddle pulmonary embolism. Our patient's blood pressure remained stable however. The intensive care team was consulted, accepted the patient to the unit, and then arranged for transfer to a tertiary care cardiothoracic center for possible surgical intervention. Echocardiogram (Figures 3 and 4 and Video 1 in Supplementary Material available online at https://doi.org/10.1155/2017/6752709) that morning remarkably displayed whirling echogenic mass in the atria and McConnell's sign.

**Figure 5:**
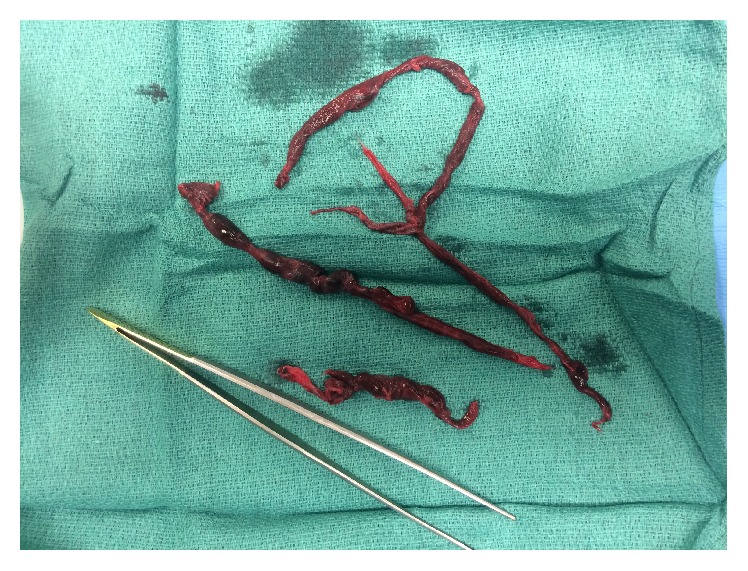
Clot retrieved at embolectomy.

**Figure 6:**
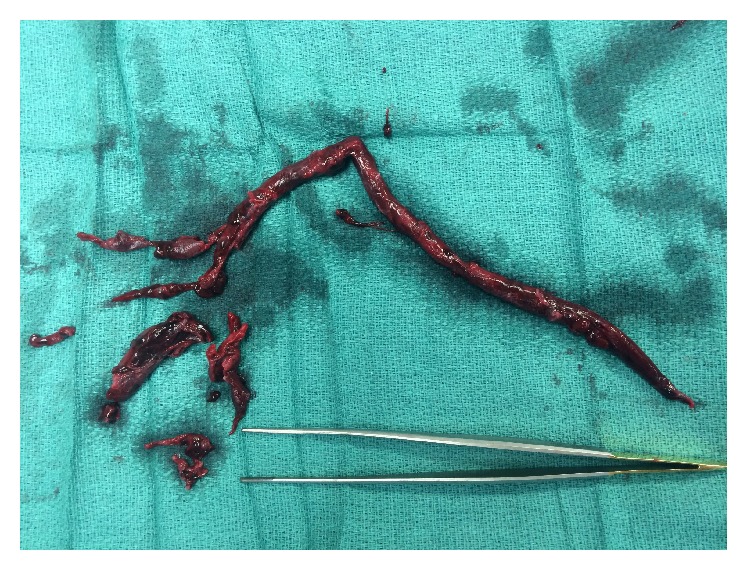
Clot retrieved at embolectomy.

He arrived at the tertiary care hospital around noon and was urgently taken to the operating theater where he underwent median sternotomy, cardiopulmonary bypass, and clamping of the aorta (which also helped to prevent systemic embolization). When the incision was made for cannulation of the inferior vena cava, a large amount of clot extruded through the incision site. The patient had right atriotomy and pulmonary endarterectomy with extraction of a large amount of thrombotic material that obstructed the pulmonary arteries (Figures 5 and 6). The PFO was sutured close in two layers. The surgery was performed without complications and the patient tolerated the procedure well.

The patient was also evaluated by the Hematology team for hypercoagulable state and thrombophilic screen was sent off. This included Factor V Leiden, protein C, protein S, lupus anticoagulant, anticardiolipin, prothrombin gene mutation, and beta-2 glycoprotein. All of these have returned negative.

## 3. Discussion

Our patient endorsed a relatively sedentary lifestyle at baseline, with recent hospitalization and increased immobilization after incision and drainage of the MRSA left foot abscess likely contributing to a greater risk of thrombogenesis. There have been other cases described in the literature of lower limb infection leading to DVT/PE, some of which also involved MRSA [1, 2]. A lower limb Doppler was not done in this case; however, in retrospect a left lower extremity DVT was the likely origin of the clot. Given the extent of thrombosis in this otherwise well young man, it is also reasonable to assume that there is some underlying thrombophilia, but thrombophilic workup has so far been unremarkable.

The visualization of a right atrial thrombus is referred to as a thrombus in transit and is a very rare echocardiographic finding, carrying a high mortality rate of up to 29% [3, 4]. It is even more rare to visualize a thrombus across PFO [5, 6]. This is particularly unique in such young patient as the average age for similar presentation is 58 ± 16 years (our patient is 25 years old). Death due to cardiogenic shock and/or right heart failure has been noted in 44% of patients who presented with an embolus straddling the PFO and death due to stroke in nearly 16% [7].

Our patient presented with acute shortness of breath and was discovered to have saddle PE with massive clot burden and thrombus-in-transit straddling PFO: a dangerous diagnosis. Although the rarity of this diagnosis results in a paucity of standardized treatment guidelines, studies have shown improved survival with surgical embolectomy as compared to anticoagulation alone in patients who are hemodynamically stable with low to moderate surgical risk [8, 9]. Thrombolysis, while used for patients in higher risk groups, may be associated with higher rates of mortality [10, 11]. In accordance with these studies and suggested treatment algorithm our patient underwent urgent embolectomy and PFO closure and was started on anticoagulation postoperatively. He suffered no neurological event and did well with aggressive surgical management.

## Supplementary Material

Large thrombus whirling within the right atrium and crossing the tricuspid valve into the right ventricle in diastole. The right ventricle is significantly dilated and dysfunction and a McConnell's sign is present. An atrial septal defect can also be appreciated.

## Figures and Tables

**Table 1:** Laboratory results.

Test	Result	Ref. range	Test	Result	Ref. range
ABG on room air			WBC (×10^9^/L)	10.9	(4.5–11.5)
pH	7.45	(7.35–7.45)	Hemoglobin (g/dL)	14.4	(14–18)
pO_2_ (mmHg)	67	(80–100)	Platelet (×10^9^/L)	221	(150–450)
pCO_2_ (mmHg)	27.6	(35–45)	PT (sec)	11.4	(9.25–12.35)
HCO_3_ (mEq/L)	19	(22–28)	PTT (sec)	22.8	(25.0–33.9)
sO_2_	93.8%	(92–100)	D-dimer (ng/mL)	37,529	(0–500)
Lactate (mmol/L)	1.2	(0–2)	Troponin I (ng/mL)	0.395	(0–0.045)
Glucose (mmol/L)	244	(70–99)	BNP (pg/mL)	95.1	(0–100)

## Abbreviations

ABG: Arterial blood gas
BNP: Brain-type natriuretic peptide
DVT: Deep venous thrombosis
PE: Pulmonary embolism
PFO: Patent foramen ovale
PT: Prothrombin time
aPTT: Activated partial thromboplastin time
MRSA: Methicillin-resistant* Staphylococcus aureus*
WBC: White blood cell count.
